# Interpreting polygenic scores, polygenic adaptation, and human phenotypic differences

**DOI:** 10.1093/emph/eoy036

**Published:** 2018-12-27

**Authors:** Noah A Rosenberg, Michael D Edge, Jonathan K Pritchard, Marcus W Feldman

**Affiliations:** 1 Department of Biology, Stanford University, Stanford, CA, USA; 2 Department of Evolution and Ecology, University of California, Davis, CA, USA; 3 Howard Hughes Medical Institute, Stanford University, Stanford, CA, USA; 4 Department of Genetics, Stanford University, Stanford, CA, USA

**Keywords:** adaptation, health disparities, human variation, polygenic scores, population genetics

## Abstract

Recent analyses of polygenic scores have opened new discussions concerning the genetic basis and evolutionary significance of differences among populations in distributions of phenotypes. Here, we highlight limitations in research on polygenic scores, polygenic adaptation and population differences. We show how genetic contributions to traits, as estimated by polygenic scores, combine with environmental contributions so that differences among populations in trait distributions need not reflect corresponding differences in genetic propensity. Under a null model in which phenotypes are selectively neutral, genetic propensity differences contributing to phenotypic differences among populations are predicted to be small. We illustrate this null hypothesis in relation to health disparities between African Americans and European Americans, discussing alternative hypotheses with selective and environmental effects. Close attention to the limitations of research on polygenic phenomena is important for the interpretation of their relationship to human population differences.

## INTRODUCTION

We are currently witnessing a surge in public interest in the intersection of evolutionary genetics with such topics as cognitive phenotypes, disease, race and heritability of human traits [1–7]. This attention emerges partly from recent advances in genomics, including the introduction of polygenic scores—the aggregation of estimated effects of genome-wide variants to predict the contribution of a person’s genome to a phenotypic trait [8–10]—and a new focus on polygenic adaptations, namely adaptations that have occurred by natural selection on traits influenced by many genes [11–13].

Theories involving natural selection have long been applied in the scientific literature to explain mean phenotypic differences among human populations [14–16]. Although new tools for statistical analysis of polygenic variation and polygenic adaptation provide opportunities for studying human evolution and the genetic basis of traits, they also generate potential for misinterpretation. In the past, public attention to research on human variation and its possible evolutionary basis has often been accompanied by claims that are not justified by the research findings [17]. Recognizing pitfalls in the interpretation of new research on human variation is therefore important for advancing discussions on associated sensitive and controversial topics.

## COMPLEX PHENOTYPES AND POLYGENIC SCORES

Over the past 15 years, genomic analyses have identified thousands of genetic variants that contribute statistically to variation in complex phenotypes, traits that have complex patterns of inheritance and that are affected by large numbers of genes in combination with environmental factors [18–20]. In a typical genomic study of a complex human phenotype—a genome-wide association study (GWAS)—genotypes at thousands or millions of sites across the human genome are each tested in a sample of people for statistical association with the phenotype. Each variant identified by such a study as statistically associated with the phenotype can be assigned an effect size, representing the estimated magnitude of the increase in the trait (for quantitative phenotypes) or risk or liability for the trait (for binary phenotypes) that is associated with possession of a copy of the variant.

For many complex phenotypes, identification and analysis of contributing genomic variants—most having small phenotypic effects—has led to the formulation of polygenic scores, quantities that seek to predict a trait value associated with a specific genome-wide set of genotypes [10]. For a quantitative phenotype, a polygenic score for an individual genome represents an aggregation, usually in the form of a sum, of the estimated effect sizes of the genetic variants in the genome (Table 1). Polygenic score estimation of underlying genetic propensities typically proceeds from GWAS outcomes.

**Table 1.** Key concepts as used in this study

Term	Meaning
Apportionment of genetic diversity	A calculation that divides genetic variation seen among individuals into components due to differences among individuals from the same population and due to differences among different populations
Binary phenotype	A phenotype that takes on one of two states, such as presence or absence of a disease
Complex phenotype	A phenotype that has a complex inheritance pattern within families and that is generally affected by many genes as well as environmental factors
Directional selection	Natural selection that favors a change in the value of a quantitative phenotype in a specific direction, either up or down
Divergent selection	Natural selection that for a quantitative phenotype acts to magnify the difference in the phenotype between a pair of populations
Effect size	The magnitude of the increase in a trait that is associated with possession of a copy of a specific genetic variant
Gene-environment interaction	A situation in which the contribution of the genotype to the phenotype depends on the environment
Genome-wide association study (GWAS)	A study in which alleles at sites spread across the genome are each tested for statistical association with a phenotype
Heritability	The fraction of phenotypic variance explained by genetic variation in the context of a specific range of environmental variation
Linkage disequilibrium	The correlation between alleles at separate genomic sites
Neutral model	A model of population-genetic forces in which no selection occurs, so that no genotype is favored or disfavored
Polygenic adaptation	Adaptation that has occurred by natural selection on traits influenced by a large number of genes
Polygenic score	An aggregate value that represents an estimated contribution of a genome to a phenotype and that can be viewed as an estimate of an underlying genetic propensity
Quantitative phenotype	A phenotype that varies on a quantitative scale rather than being either present or absent

Polygenic scores have provided new tools for interpreting human genomes in the setting of complex phenotypes for which effect sizes of genetic variants are small. For example, they have contributed new approaches to risk prediction for adverse phenotypes related to heart disease [21–23]. Using polygenic scores, it is now possible to combine information from millions of genomic variants to identify people whose overall polygenic risk of coronary artery disease is as high as that of patients with monogenic lipid disorders such as familial hypercholesterolemia [23]. Although for many genetically complex phenotypes, polygenic scores currently explain too small a fraction of variation in the phenotype to be clinically meaningful, such risk calculations contribute to the promise of the genomic era to produce actionable predictions about complex phenotypes on the basis of the accumulation of many small genomic contributions [24].

## GENETIC BASIS OF POPULATION DIFFERENCES IN COMPLEX PHENOTYPES

The genetic underpinnings of population differences in phenotype distributions have been of perennial interest in human genetics, and the use of polygenic scores promises to generate progress in understanding phenotypic differences among populations. However, interpretation of such differences in relation to polygenic score differences requires careful analysis. Although distributions of individual-level polygenic scores might differ among populations, differences in these distributions might have many potential causes, and might or might not reflect meaningful biological phenomena.

**Figure 1.**
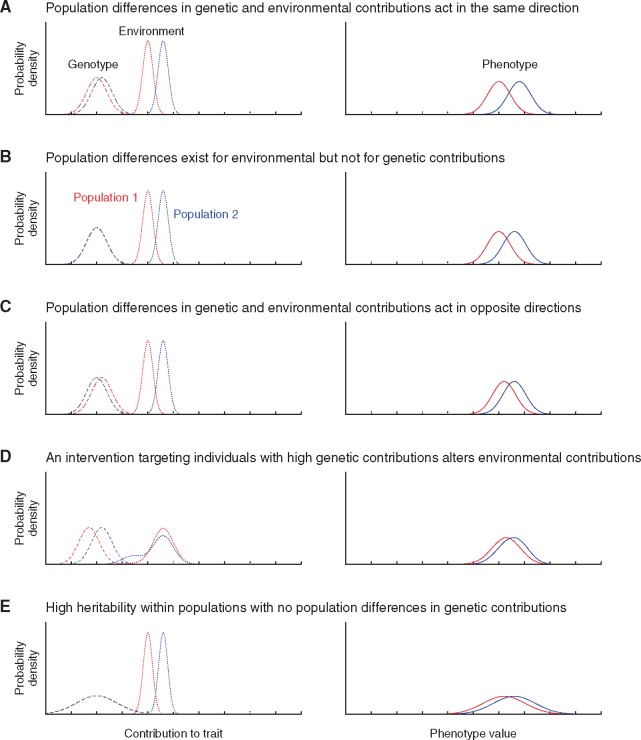
The contribution of polygenic score distributions to phenotype distributions. Two populations are considered, populations 1 (red) and 2 (blue). Each population has a distribution of genetic propensities, which are treated as accurately estimated in the form of polygenic scores (left). The genetic propensity distribution and an environment distribution sum to produce a phenotype distribution (right). All plots have the same numerical scale. **(A)** Environmental differences amplify an underlying difference in genetic propensities. **(B)** Populations differ in their phenotypes despite having no differences in genetic propensity distributions. **(C)** Environmental differences obscure a difference in genetic propensities opposite in direction to the difference in phenotype means. **(D)** Similarity in phenotype distributions is achieved despite a difference in genetic propensity distributions by an intervention that reduces the environmental contribution for individuals with polygenic scores above a threshold. **(E)** Within populations, heritability is high, so that genetic variation explains the majority of phenotypic variation; however, the difference between populations is explained by an environmental difference. Panels (A–C and E) present independent normal distributions for genotype and environment that sum to produce normal distributions for phenotype. In **(D)**, (genotype, environment) pairs are simulated from independent normal distributions and a negative constant—reflecting the effect of a medication or other intervention—is added to environmental contributions associated with simulated genotypic values that exceed a threshold

The main novelty in analyses of polygenic score distributions among populations is that many trait-associated genetic variants that were previously unknown are now known. Earlier studies of the role of genetics in phenotypic differences among populations often relied on statistics such as heritabilities—fractions of phenotypic variance explained by genetic variation [25]—which require no knowledge of contributing genetic variants. Although estimates of the contributions of specific genetic variants advance modern analyses beyond classical heritability statistics, many of the pre-genomic-era limitations on the use of heritability to make inferences about the genetic basis of phenotypic differences among populations [26–28] continue to apply, in updated form (Fig. 1). Limitations in the interpretation of polygenic score differences can be of two kinds: those due to the manner in which genes and environment contribute to traits, irrespective of statistical issues involved in estimating the contributions from data, and those due to statistical phenomena in the estimation process.

### Conceptual limitations

First, population differences in environmental factors are important for interpreting population differences in a phenotype distribution. Depending on environmental contributions, a difference in mean phenotype might or might not reflect a difference in the magnitude of genetic effects among populations; population differences in phenotype distributions do not reveal which population has greater genetic propensities on average, whether the observed difference would persist if the environment were altered, or even whether a difference in genetic propensities exists at all (Fig. 1A–C). That polygenic scores ignore the role of environmental influences on phenotype is particularly relevant when the phenotype can be readily modified, as in the use of statins as medications to control the lipid levels that contribute to coronary artery disease risk. In such cases, a difference in polygenic score—though possibly a genuine reflection of underlying genetic propensities—might be incorrectly inferred to represent an unchangeable genetic difference among populations rather than one that can be altered by an environmental change (Fig. 1D). Instead, the potential for significant modification of the environmental contribution renders polygenic score differences between populations largely unconnected to population differences in phenotype distributions.

Second, gene–gene and gene–environment interactions influence traits, meaning that associations between specific genotypes and a phenotype—and the importance of the genetic contributions—might differ among populations with different allele frequencies or distributions of environmental variables. In other words, because the contributions of genomic variants can differ among populations due to interactions with other variants and with environmental variables, the effects of a variant on a trait can have different magnitudes in different populations, and effects of multiple variants in one or more genes can combine in different ways. Large population differences in disease risk for well-known alleles such as *APOE*-ɛ4 in Alzheimer disease [29, 30] highlight the challenge of determining how population differences in effect size might be affected by interaction effects.

Finally, mean differences between populations in polygenic scores might not be informative for prediction about phenotype differences between randomly chosen people from a pair of populations if polygenic score distributions have substantial overlap (Fig. 1A, C and D). In such cases, predictive potential is limited even if a difference in population means is seen to be statistically significant in the large sample sizes typical of genome-wide association studies.

### Statistical limitations

Beyond the conceptual challenges, which are inherent in interpreting population differences in polygenic scores, the process of estimating a difference in the scores themselves is subject to additional limitations. Genotypic effects estimated only in one population might not apply to other populations for a number of reasons. Effect estimates might rely on sites that were ascertained for variability in one set of populations and whose systematic differences in allele frequencies between populations contribute to systematic biases in polygenic score estimates in other populations [31]. These estimates might also fail to consider many sites variable only in those other populations.

A third limit to transferability of effect estimates arises from population differences in features of correlations between nearby sites—linkage disequilibrium patterns—that influence aggregations of signals across loci [32]. So far, because most genome-wide association studies have been conducted in populations with European ancestry [33], the effect sizes used in calculating polygenic scores have been calibrated on Europeans, and their values might not transfer accurately to other populations. Even among populations with European ancestry, subtle ancestry differences between samples can lead to polygenic scores that overstate between-population differences: small biases in locus-wise effect estimates that arise from the ancestry differences can potentially accumulate across loci [34, 35].

### Summary

These limitations illustrate that much of the complexity embedded in use of polygenic scores—the effects of the environment on phenotype and its relationship to genotype, the proportion of variance explained, and the peculiarities of the underlying GWAS data that have been used to estimate effect sizes—is obscured by the apparent simplicity of the single values computed for each individual for each phenotype. Consequently, in using polygenic scores to describe genomic contributions to traits, particularly traits for which the total contribution of genetic variation to trait variation, as measured by heritability, is low—but even if it is high (Fig. 1E)—a difference in polygenic scores between populations provides little information about potential genetic bases for trait differences between those populations.

Unlike heritability, which ranges from 0 to 1 and therefore makes it obvious that the remaining contribution to phenotypic variation is summarized by its difference from 1, the limited explanatory role of genetics is not embedded in the nature of the polygenic scores themselves. Although polygenic scores encode knowledge about specific genetic correlates of trait variation, they do not change the conceptual framework for genetic and environmental contribution to population differences. Attributions of phenotypic differences among populations to genetic differences should therefore be treated with as much caution as similar genetic attributions from heritability in the pre-genomic era.

## POLYGENIC ADAPTATION

Genomic investigations have provided insights into how natural selection has given rise to differences in phenotypes that vary geographically, such as skin pigmentation, lactase persistence and altitude-related physiology [14–16]. Success in these well-known examples, each involving natural selection primarily on one or a few genes of large effect, has encouraged the search for other phenotypes that might have experienced different histories of natural selection in different populations. Recent interest focuses on traits such as height [12, 36, 37] that are influenced by large numbers of genetic variants, each having a small effect on the trait, and that lend themselves to analysis with polygenic scores.

### The null expectation

Evidence that natural selection has contributed to population differences in some specific traits can invite claims that it has also influenced phenotypic differences and underlying genetic differences in other traits. It might be hypothesized, for example, that a population with a higher mean trait value has experienced selection favoring the high value, and perhaps also that selection has favored a lower value in a second population. This type of hypothesis might entail that the difference in phenotype distributions in Fig. 1A–C results from genetic propensity differences between populations that follow the same direction as the phenotype, as in Fig. 1A but not in Fig. 1B or C, and that those distributions reflect natural selection rather than selectively neutral evolutionary processes. The hypothesis might appear to derive support from the fact that sufficient genetic variation exists among populations to infer the ancestral populations of individual genomes at a local geographical scale [38–40], and the genetic differences evident from ancestry inferences might then be attributed to natural selection. However, the inference from the existence of differences in trait distributions between two populations that natural selection has acted to produce genetic differences between those populations requires several careful steps [41].

One key component of the inference of polygenic adaptation is the use of an appropriate null expectation for polygenic score distributions and phenotypic differences [12, 42]. In deriving such an expectation, an important insight from selectively neutral population-genetic models is that irrespective of the number of genetic loci contributing to a polygenic trait, the expected difference among populations in the trait is predicted to have comparable magnitude to the classical estimate of the ‘apportionment of human genetic diversity’, the extent of human genetic difference at a single randomly chosen polymorphic genetic locus [12, 43, 44]. In other words, analogous measures of population differences in quantitative phenotypic traits and genetic loci—termed *Q*_ST_ and *F*_ST_, respectively—are approximately equal in neutral evolutionary models that include genetic drift but not natural selection. Because many loci contribute to a quantitative trait, and each locus experiences the same random process of genetic drift independent of the size and direction of its trait contribution, phenotypic differences among populations are predicted under neutrality to be similar in magnitude to typical genetic differences among populations.

The genetic apportionment computation shows that genetic differences among populations, as measured by *F*_ST_, are small in comparison with variation within populations [45–47]. Although the among-population variation suffices to infer ancestral populations from individual genomes, analysis of models for the genetic basis of phenotypes finds that under neutrality, the magnitude of phenotypic differences connects to the apportionment computation rather than the ancestry computation [42, 44].

### Selection or environmental effects?

Departures from the null expectation for phenotypic differences among populations can be due to a combination of (i) population differences in environmental influences on phenotypes, and (ii) differential natural selection among populations that generates a substantial population difference in polygenic score distributions. However, only with strong directional selection on a trait in one population, or strong directional selection in opposite directions in a population pair, is a phenotypic difference between populations attributable largely to natural selection. In other words, because of environmental effects, the difference in phenotype distributions in Fig. 1A–C need not reflect a parallel difference in genetic propensities as in Fig. 1A, but rather no difference as in Fig. 1B or a difference opposite in direction as in Fig. 1C; even a parallel difference as in Fig. 1A might reflect a neutral expectation rather than natural selection, possibly amplified by environmental effects.

**Figure 2.**
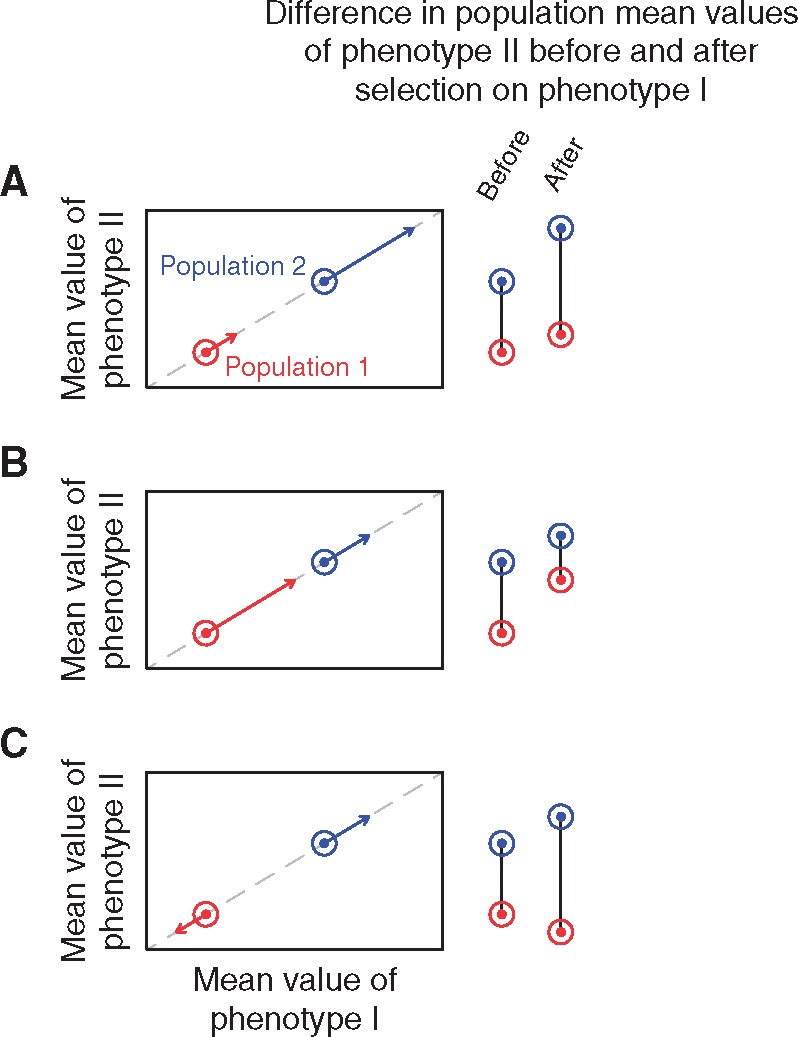
The change in mean phenotypic differences between populations resulting from natural selection acting on a correlated phenotype. In the graphs, two populations with different trait values, indicated by circles, experience natural selection, indicated by arrows. Selection acts on phenotype I, which is strongly positively correlated with phenotype II. The action of selection changes the population difference in the mean values of phenotype II, as indicated to the right of the graphs. **(A)** Directional selection on phenotype I in the same direction in the two populations increases the population difference for phenotype II. **(B)** Directional selection on phenotype I in the same direction in the two populations decreases the population difference for phenotype II. **(C)** Divergent natural selection on phenotype I increases the population difference for phenotype II

Trait correlations can also complicate inferences of selection differences, as a scenario in which differences in polygenic score distributions among populations parallel differences for a specific phenotype might be due not only to environmental factors, but instead to natural selection on other correlated traits [48]. Selection on a correlated trait might occur in different directions in a pair of populations or with different magnitudes in the same direction, and therefore need not increase genetic differences between populations in the way that divergent selection for the initial trait might suggest (Fig. 2).

For these reasons, an observed between-population difference in phenotype distributions is not easily ascribed to divergent selection. Indeed, a challenge is to establish whether polygenic adaptation has even occurred. In within-population polygenic adaptation tests, for loci across the genome, GWAS-based locus effect sizes are considered with selection signals estimated for those loci. An aggregate signal of positive selection at loci with large effect sizes is taken to suggest that selection has inflated the frequencies of alleles contributing to the trait, so that the trait has undergone polygenic adaptation. Recent studies of height have suggested that polygenic adaptation tests are sensitive to the choice of GWAS data that provide the effect sizes: even if two sets of effect sizes produce correlated polygenic scores, effect sizes estimated from one study can generate erroneously exaggerated signatures of polygenic adaptation when assessing polygenic adaptation in a second dataset [34, 35]. This result, which arises from subtle population differences between study samples, calls into question claims about polygenic adaptation even of traits for which it has been most extensively investigated.

### Summary

To date, strong effects of directional selection on human population differences have been verifiable primarily for traits connected to predictable categories of geographic variability, including dietary adaptations, infectious disease resistance and skin pigmentation [14–16]. As speculations about features of natural selection in human populations proliferate, hypotheses about selection on specific phenotypes should not be treated as being as plausible *a priori* as a general null population-genetic model of phenotypic similarity among populations. Dramatic claims about divergent selection should continue to be regarded cautiously in the absence of strong quantitative evidence.

## THE CASE OF HEALTH DISPARITIES

Health disparities between African Americans and European Americans in the USA provide a useful case for examining genetic and environmental contributions to phenotypic differences among populations. In a study of African Americans and European Americans (treated as socially rather than genetically defined populations), among 36 physiologically diverse causes of death, adjusting for other factors, African Americans lost more years of life than European Americans in 28 of the 36 [49]. In the simplest null model in which many genes contribute to a trait chosen at random, with no directional selection, each of a pair of groups has probability 0.5 of having the larger mean value for the trait. In this model, systematic health disparities are unlikely: assuming that no genetic correlation exists between phenotypic outcomes, the binomial probability that the trait value is larger in one of a pair of populations for at least 28 of 36 independent phenotypes is 0.0012 [42].

It might be proposed that different strengths of directional selection have contributed to the population difference between African Americans and European Americans. However, a related computation of the overall influence of natural selection in human history, relying on measures of selection against deleterious variants rather than directional selection of favorable variants, does not suggest strong systematic differences in the magnitude of selection among different continental population groups [50–52]; indeed, some researchers have argued for a greater level of deleterious variation in non-Africans rather than in Africans [50, 52], a pattern opposite to what might be expected given the direction of health disparities.

Whereas natural selection cannot easily explain the observed population difference, systematic environmental effects that contribute to an increase in non-genetic risk factors in African Americans—current and historical racism, for instance [53–55]—could, on the other hand, explain such marked differences. This example of health disparities illustrates important features of reasoning in a manner informed by population genetics about the extent to which phenotypic differences can be assigned to genetic differences among populations, and to natural selection: a selectively neutral null model, an awareness of environmental factors and a simultaneous analysis of multiple traits.

## PROSPECTS

With ongoing discoveries in human genomics, it is becoming possible to address topics concerning the genetic and phenotypic differences among populations that have been the subject of much speculation. Recent advances are sure to lead to proliferation of widely disseminated hypotheses about polygenic scores, population differences and natural selection. Unfortunately, history suggests that multiple forms of misrepresentation of findings in human genetics can lend the authority of science to claims that the underlying research does not validate and might actively contradict.

One recurring problem in the dissemination of studies of human genetic differences within and beyond the scientific community is the attribution of interpretive weight to plausibly compelling hypotheses about natural selection in spite of a lack of evidence [56]. Other problems include reliance in scientists’ publicity materials and in news reports on racialized language and exaggerated views of race as biological [57], when modern discourse in population genetics instead uses non-racial conceptual structures for characterizing and analyzing human variation [58]. Miscalibration of news coverage—not to the magnitude of advances but rather to the greater public appetite for new developments in controversial areas of genetics [59]—can result in cascading distortions of the genetic basis of phenotypic traits that studies do not imply and that their authors do not support [17].

As developments on polygenic scores and polygenic adaptation connect closely to topics that have long been of central interest in human evolutionary genetics, the field can provide context for the emerging plethora of results relevant to interpretations of the roles of genetics and natural selection in contributing to traits; limitations of interpretations of research in new directions are not restricted to the topics emphasized here [41, 60]. Vigilance in promoting careful and evidence-supported explanations and in clarifying the caveats that affect ongoing genetic research programs continues to be required both from investigators and from those who disseminate the findings.

## References

[1] Regalado A. Forecasts of genetic fate just got a lot more accurate. *MIT Technology Review* 2018 https://www.technologyreview.com/s/610251/forecasts-of-genetic-fate-just-got-a-lot-more-accurate/ (21 February 2018, date last accessed).

[2] Evans G. The unwelcome revival of ‘race science.’ *The Guardian.* 2018 https://www.theguardian.com/news/2018/mar/02/the-unwelcome-revival-of-race-science (2 March 2018, date last accessed).

[3] Reich D. How genetics is changing our understanding of ‘race’. *New York Times.* 2018 https://www.nytimes.com/2018/03/23/opinion/sunday/genetics-race.html (23 March 2018, date last accessed).

[4] Kahn J, Nelson A, Graves JL et al How not to talk about race and genetics. BuzzFeed. 2018 https://www.buzzfeed.com/bfopinion/race-genetics-david-reich? utm_term=.xqzaXqD4K#.ev1O9xeQm (30 March 2018, date last accessed).

[5] Regalado A. DNA tests for IQ are coming, but it might not be smart to take one. *MIT Technology Review* 2018 https://www.technologyreview.com/s/610339/dna-tests-for-iq-are-coming-but-it-might-not-be-smart-to-take-one/ (2 April 2018, date last accessed).

[6] Harden P. Genetic test scores predicting intelligence are not the new eugenics. Leapsmag. 2018 https://leapsmag.com/genetic-test-scores-predicting-intelligence-are-not-the-new-eugenics/ (13 April 2018, date last accessed).

[7] Zimmer C. (2018) She Has Her Mother’s Laugh. New York: Dutton.

[8] International Schizophrenia Consortium Common polygenic variation contributes to risk of schizophrenia and bipolar disorder. Nature 2009;460:748–52. 19571811 10.1038/nature08185 PMC3912837

[9] Dudbridge F. Power and predictive accuracy of polygenic risk scores. PLoS Genet 2013;9:e1003348. 23555274 10.1371/journal.pgen.1003348 PMC3605113

[10] Wray NR, Lee SH, Mehta D et al Polygenic methods and their application to psychiatric traits. J Child Psychol Psyc 2014;55:1068–87. 10.1111/jcpp.12295 25132410

[11] Pritchard JK, Pickrell JK, Coop G. The genetics of adaptation: hard sweeps, soft sweeps, and polygenic adaptation. Curr Biol 2010;20:R208–15. 20178769 10.1016/j.cub.2009.11.055 PMC2994553

[12] Berg JJ, Coop G. A population genetic signal of polygenic adaptation. PLoS Genet 2014;10:e1004412. 25102153 10.1371/journal.pgen.1004412 PMC4125079

[13] Racimo F, Berg JJ, Pickrell JK. Detecting polygenic adaptation in admixture graphs. Genetics 2018;208:1565–84. 29348143 10.1534/genetics.117.300489 PMC5887149

[14] Novembre J, Di Rienzo A. Spatial patterns of variation due to natural selection in humans. Nat Rev Genet 2009;10:745–55. 19823195 10.1038/nrg2632 PMC3989104

[15] Lachance J, Tishkoff SA. Population genomics of human adaptation. Annu Rev Ecol Evol Syst 2013;44:123–43. 25383060 10.1146/annurev-ecolsys-110512-135833 PMC4221232

[16] Jeong C, Di Rienzo A. Adaptations to local environments in modern human populations. Curr Opin Genet Dev 2014;29:1–8. 25129844 10.1016/j.gde.2014.06.011 PMC4258478

[17] Beckwith J. The persistent influence of failed scientific ideas In: Krimsky S, Gruber J (eds.). Genetic Explanations: Sense and Nonsense. Cambridge, MA: Harvard University Press, 2013, 173–85.

[18] Stranger BE, Stahl EA, Raj T. Progress and promise of genome-wide association studies for human complex trait genetics. Genetics 2011;187:367–83. 21115973 10.1534/genetics.110.120907 PMC3030483

[19] Welter D, MacArthur J, Morales J et al The NHGRI GWAS Catalog, a curated resource of SNP-trait associations. Nucleic Acids Res 2014;42:D1001–6. 24316577 10.1093/nar/gkt1229 PMC3965119

[20] Visscher PM, Wray NR, Zhang Q et al 10 years of GWAS discovery: biology, function, and translation. Am J Hum Genet 2017;101:5–22. 28686856 10.1016/j.ajhg.2017.06.005 PMC5501872

[21] Kullo IJ, Jouni H, Austin EE et al Incorporating a genetic risk score into coronary heart disease risk estimates. Circulation 2016;133:1181–8. 26915630 10.1161/CIRCULATIONAHA.115.020109 PMC4803581

[22] Paquette M, Chong M, Thériault S et al Polygenic risk score predicts prevalence of cardiovascular disease in patients with familial hypercholesterolemia. J Clin Lipidol 2017;11:725–32. 28456682 10.1016/j.jacl.2017.03.019

[23] Khera AV, Chaffin M, Aragam KG et al Genome-wide polygenic scores for common diseases identify individuals with risk equivalent to monogenetic mutations. Nat Genet 2018;50:1219–24. 30104762 10.1038/s41588-018-0183-z PMC6128408

[24] Torkamani A, Wineinger NE, Topol EJ. The personal and clinical utility of polygenic risk scores. Nat Rev Genet 2018;19:581–90. 29789686 10.1038/s41576-018-0018-x

[25] Visscher PM, Hill WG, Wray NR. Heritability in the genomics era—concepts and misconceptions. Nat Rev Genet 2008;9:255–66. 18319743 10.1038/nrg2322

[26] Lewontin RC. The analysis of variance and the analysis of causes. Am J Hum Genet 1974;26:400–11. 4827368 PMC1762622

[27] Feldman MW, Lewontin RC. The heritability hang-up. Science 1975;190:1163–8. 1198102 10.1126/science.1198102

[28] Feldman MW, Ramachandran S. Missing compared to what? Revisiting heritability, genes and culture. Philos Trans R Soc Lond B 2018;373:20170064. 29440529 10.1098/rstb.2017.0064 PMC5812976

[29] Tang M-X, Stern Y, Marder K et al The *APOE*-ɛ4 allele and the risk of Alzheimer disease among African Americans, whites, and Hispanics. J Am Med Assoc 1998;279:751–5. 10.1001/jama.279.10.751 9508150

[30] Rajabli F, Feliciano BE, Celis K et al Ancestral origin of *ApoE* ɛ4 Alzheimer disease risk in Puerto Rican and African American populations. PLoS Genet 2018;14:e1007791. 30517106 10.1371/journal.pgen.1007791 PMC6281216

[31] Kim MS, Patel KP, Teng AK et al Genetic disease risks can be misestimated across global populations. Genome Biol 2018;19:179. 30424772 10.1186/s13059-018-1561-7 PMC6234640

[32] Martin AR, Gignoux CR, Walters RK et al Human demographic history impacts genetic risk prediction across diverse populations. Am J Hum Genet 2017;100:635–49. 28366442 10.1016/j.ajhg.2017.03.004 PMC5384097

[33] Popejoy AB, Fullerton SM. Genomics is failing on diversity. Nature 2016;538:161–4. 27734877 10.1038/538161a PMC5089703

[34] Berg JJ, Harpak A, Sinnott-Armstrong N et al Reduced signal for polygenic adaptation of height in UK Biobank. bioRxiv 2018; doi: 10.1101/354951. 10.7554/eLife.39725 PMC6428572 30895923

[35] Sohail M, Maier RM, Ganna A et al Signals of polygenic adaptation on height have been overestimated due to uncorrected population structure in genome-wide association studies. bioRxiv 2018; doi: 10.1101/355057. 10.7554/eLife.39702 PMC6428571 30895926

[36] Turchin MC, Chiang CWK, Palmer CD et al Evidence of widespread selection on standing variation in Europe at height-associated SNPs. Nat Genet 2012;44:1015–9. 22902787 10.1038/ng.2368 PMC3480734

[37] Field Y, Boyle EA, Telis N et al Detection of human adaptation during the past 2000 years. Science 2016;354:760–4. 27738015 10.1126/science.aag0776 PMC5182071

[38] Jakobsson M, Scholz SW, Scheet P et al Genotype, haplotype and copy-number variation in worldwide human populations. Nature 2008;451:998–1003. 18288195 10.1038/nature06742

[39] Li JZ, Absher DM, Tang H et al Worldwide human relationships inferred from genome-wide patterns of variation. Science 2008;319:1100–4. 18292342 10.1126/science.1153717

[40] Novembre J, Peter BM. Recent advances in the study of fine-scale population structure in humans. Curr Opin Genet Dev 2016;41:98–105. 27662060 10.1016/j.gde.2016.08.007 PMC5291306

[41] Novembre J, Barton NH. Tread lightly interpreting polygenic tests of selection. Genetics 2018; 208:1351–5. 29618592 10.1534/genetics.118.300786 PMC5886544

[42] Edge MD, Rosenberg NA. A general model of the relationship between the apportionment of human genetic diversity and the apportionment of human phenotypic diversity. Hum Biol 2015;87:313–37. 27737590 10.13110/humanbiology.87.4.0313 PMC8504698

[43] Leinonen T, McCairns RJS, O’Hara RB, Merilä J. Q_ST_–F_ST_ comparisons: evolutionary and ecological insights from genomic heterogeneity. Nat Rev Genet 2013;14:179–90. 23381120 10.1038/nrg3395

[44] Edge MD, Rosenberg NA. Implications of the apportionment of human genetic diversity for the apportionment of human phenotypic diversity. Stud Hist Philos Biol Biomed Sci 2015;52:32–45. 25677859 10.1016/j.shpsc.2014.12.005 PMC4516610

[45] Lewontin RC. The apportionment of human diversity. Evol Biol 1972;6:381–98.

[46] Barbujani G, Magagni A, Minch E, Cavalli-Sforza LL. An apportionment of human DNA diversity. Proc Natl Acad Sci USA 1997;94:4516–9. 9114021 10.1073/pnas.94.9.4516 PMC20754

[47] Rosenberg NA. A population-genetic perspective on the similarities and differences among worldwide human populations. Hum Biol 2011;83:659–84. 22276967 10.3378/027.083.0601 PMC3531797

[48] Lande R, Arnold SJ. The measurement of selection on correlated characters. Evolution 1983;37:1210–26. 28556011 10.1111/j.1558-5646.1983.tb00236.x

[49] Wong MD, Shapiro MF, Boscardin WJ, Ettner SL. Contribution of major diseases to disparities in mortality. N Engl J Med 2002;347:1585–92. 12432046 10.1056/NEJMsa012979

[50] Lohmueller KE. The distribution of deleterious genetic variation in human populations. Curr Opin Genet Dev 2014;29:139–46. 25461617 10.1016/j.gde.2014.09.005

[51] Simons YB, Turchin MC, Pritchard JK, Sella G. The deleterious mutation load is insensitive to recent population history. Nat Genet 2014;46:220–4. 24509481 10.1038/ng.2896 PMC3953611

[52] Henn BM, Botigué LR, Bustamante CD et al Estimating the mutation load in human genomes. Nat Rev Genet 2015;16:333–43. 25963372 10.1038/nrg3931 PMC4959039

[53] Paradies Y. A systematic review of empirical research on self-reported racism and health. Int J Epidemiol 2006;35:888–901. 16585055 10.1093/ije/dyl056

[54] Gravlee CC, Non AL, Mulligan CJ. Genetic ancestry, social classification, and racial inequalities in blood pressure in southeastern Puerto Rico. PLoS One 2009;4:e6821. 19742303 10.1371/journal.pone.0006821 PMC2731885

[55] Williams DR, Mohammed SA. Discrimination and racial disparities in health: evidence and needed research. J Behav Med 2009;32:20–47. 19030981 10.1007/s10865-008-9185-0 PMC2821669

[56] Kaufman JS, Hall SA. The slavery hypertension hypothesis: dissemination and appeal of a modern race theory. Epidemiology 2003;14:111–8. 12500059 10.1097/00001648-200301000-00027

[57] Condit CM. How geneticists can help reporters get their story right. Nat Rev Genet 2007;8:815–20. 17878897 10.1038/nrg2201

[58] Rosenberg NA, Edge MD. Genetic clusters and the race debates: a perspective from population genetics In: Spencer QNJ (ed.). The Race Debates from Metaphysics to Medicine. Oxford: Oxford University Press, 2019.

[59] Caulfield T, Condit C. Science and the sources of hype. Public Health Genomics 2012;15:209–17. 22488464 10.1159/000336533

[60] Wray NR, Yang J, Hayes BJ et al Pitfalls of predicting complex traits from SNPs. Nat Rev Genet 2013;14:507–15. 23774735 10.1038/nrg3457 PMC4096801

